# *Clostridioides difficile* infection epidemiology and clinical characteristics in COVID-19 pandemic

**DOI:** 10.3389/fmed.2022.953724

**Published:** 2022-08-22

**Authors:** Silvia Vázquez-Cuesta, María Olmedo, Elena Reigadas, Luis Alcalá, Mercedes Marín, Patricia Muñoz, Emilio Bouza

**Affiliations:** ^1^Department of Clinical Microbiology and Infectious Diseases, Hospital General Universitario Gregorio Marañón, Madrid, Spain; ^2^Instituto de Investigación Sanitaria Gregorio Marañón, Madrid, Spain; ^3^Department of Biochemistry and Molecular Biology, Faculty of Biology, Universidad Complutense de Madrid, Madrid, Spain; ^4^Department of Medicine, School of Medicine, Universidad Complutense de Madrid, Madrid, Spain; ^5^ESCMID Study Group for Clostridioides difficile, Basel, Switzerland; ^6^CIBER de Enfermedades Respiratorias (CIBERES CB06/06/0058), Madrid, Spain

**Keywords:** CDI, COVID-19, SARS-CoV-2, *C. difficile*, nosocomial infection, hospital-acquired

## Abstract

Information on *Clostridioides difficile* infection (CDI) in patients with COVID-19 is scarce and points to an overall decrease of episodes during the pandemic. This situation results paradoxical, as COVID-19 patients had long periods of hospital stay and high use of antibiotics. We conducted a retrospective study from January 1st 2019 to December 31st 2020 comparing the incidence of hospital-acquired episodes of CDI (HA-CDI) among patients with and without COVID-19 admitted to our institution. During the study period, there were 47,048 patient admissions in 2019, 35,662 admissions of patients without COVID-19 in 2020 and 6,763 of COVID-19 patients. There were 68 episodes of HA-CDI in COVID-19 patients (14.75/10,000 days), 159 in 2020-non-COVID-19 patients (5.54/10,000 days) and 238 in 2019 (6.80/10,000 days). Comparison of HA-CDI in COVID-19 and non-COVID-19 patients indicates it occurs more frequently, in terms of CDI disease severity, COVID-19 does not seem to have a negative impact.

## Introduction

The COVID-19 pandemic has imposed unreasonable workload on hospitals worldwide and has forced health professionals to work under unsuitable conditions (1, 2).

A high percentage of COVID-19 patients requiring hospitalization are admitted because of pneumonia -sometimes rapidly progressive-. Fear of concomitant presence of bacterial pathogens has led to massive use of antibacterial agents (3).

Under those conditions, seems logical to expect a considerable increase in the incidence of nosocomial *Clostridioides difficile* infection (HA-CDI) (4), although preliminary data seem to *paradoxically* indicate otherwise (5–8).

Data on purported HA-CDI decrease usually consider the figures of the whole hospital population and not the incidence of HA-CDI in a specific cohort of COVID-19 patients.

Our aim was to compare the incidence of HA-CDI during 2019 and 2020 in our hospital and describe the peculiarities of this disease in the COVID-19 population.

## Materials and methods

### Setting, design, and study population

This study was carried out at Hospital General Universitario Gregorio Marañón, Madrid (Spain), a university hospital with 1,350 beds. Determination of toxigenic *C. difficile* is routinely performed on all diarrheic stool samples from patients older than 2 years.

We conducted a retrospective study between January 1, 2019 and December 31, 2020, comparing HA-CDI data in COVID-19 patients against non-COVID-19 patients.

All patients fulfilling the definition of HA-CDI were considered potential cases for this study (9).

### Definitions

An episode of CDI was defined as the presence of a positive toxigenic CDI test, accompanied by diarrhea (≥3 non-forming stools in 24 h) or findings of pseudomembranous colitis by colonoscopy, as per the definitions by the SHEA and IDSA guidelines (9). The severity of the CDI episode was defined according to the SHEA and IDSA guidelines (9).

We defined a HA-CDI when the diagnosis occurred at least 48 h after patients’ admission or documented overnight stay in a healthcare facility in the prior 4 weeks (9).

HO-CDI definition is when the diagnosis occurred at least 48 h after patients’ admission (9).

Recurrence of CDI (R-CDI) is present when CDI recurs within 8 weeks after a previous episode, provided the symptoms from the previous episode resolved after completion of initial treatment (10).

Death was considered to be CDI-related when there were no other attributable causes and/or it occurred within 10 days after CDI diagnosis and/or was due to known CDI-associated complications.

We considered immunosuppressed patients at admission those who presented with an compromised immunity due to any of the following: an active hematological malignancy (including leukemia, lymphoma, multiple myeloma), an active malignancy requiring recent cytotoxic chemotherapy, receipt of a prior hematopoietic stem cell transplant, receipt of a prior solid organ transplant, asplenia, neutropenia/pancytopenia due to other conditions, acquired or congenital immunodeficiency disorders or those receiving immunosuppressive agents.

### Microbiological testing methods

Samples were processed using a rapid detection kit for toxigenic *C. difficile*. This rapid test consists of antigen detection by immunochromatography (C Diff Quik-Chek Complete assay, TechLab, Blacksburg, VA, United States) and a real-time polymerase chain reaction (PCR) of the toxin B gene (XpertTM *C. Difficile* Assay, GeneXpert, Cepheid, Sunnyvale, CA, United States).

In addition, all samples were cultured on *C. difficile* selective agar (bioMeriéux, Marcyl’Etoile, France). Suspected toxigenic *C. difficile* colonies were confirmed using immunochromatography (C Diff Quik-Chek Complete assay, TechLab, Blacksburg, VA, United States).

For the detection of SARS-CoV-2, nasopharyngeal swabs in viral transport medium were used. Presence of SARS-CoV-2 RNA was tested by reverse transcriptase PCR. The detected genes were the N gene and Orf1a1b (Thermo Fisher^®^, Waltham, MA, United States).

### Clinical data

Patients’ age and sex (demographic data) were recorded. Regarding clinical data, the Charlson index (11) was used to quantify the degree of comorbidities. Other clinical data collected included antibiotic treatment, proton pump inhibitor use, need of nasogastric tube, mechanical ventilation, surgery, chemotherapy, or radiotherapy in the month prior to CDI diagnosis. Regarding CDI episodes, data on their severity, treatment received, treatment failure, recurrence, mortality, and CDI-related mortality were recorded.

### Statistical analysis

Data were analyzed using R (R core Team, 2020, R: A language and environment for statistical computing. R Foundation for Statistical Computing, Vienna, Austria) (12). For qualitative variables, frequencies were calculated, using 95% confidence interval for the proportions (binomial distribution). For quantitative variables, medians and interquartile ranges (IQR) or means and standard deviations (SD) were calculated.

Intergroup differences were determined using the Chi-square test. For continuous variables Student’s *t*-test or the Mann–Whitney U-test were used when normal distribution of the data could not be assumed. Normal distribution of continuous variables was tested with the Shapiro–Wilk test.

### Ethical considerations

This study was approved by the ethics committee of Hospital General Universitario Gregorio Marañón in Madrid (number MICRO.HGUGM.2021-003).

## Results

During 2019 there were a total of 47,048 admissions and during 2020 an overall of 42,445 admissions, out of which, 35,662 were non-COVID-19 and 6,763 were COVID-19 admissions to our hospital.

**FIGURE 1:**
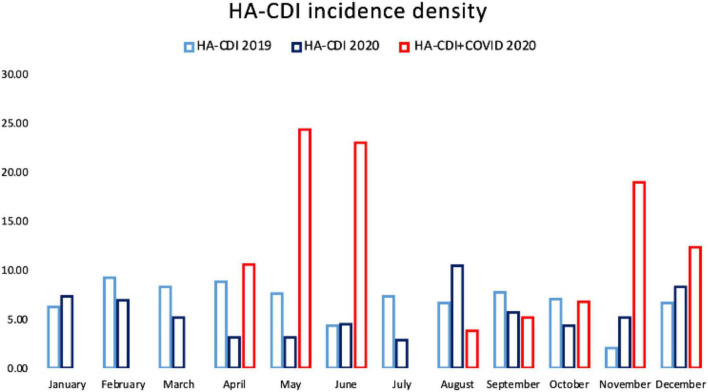
HA-CDI incidence in 2019, in 2020 in non-COVID-19 patients and in 2020 in COVID-19 patients.

The number of patients with a first episode of HA-CDI was 238, 159 and 68 among the 2019, 2020-non-COVID-19 and COVID-19 patients, respectively. HA-CDI episodes per 10,000 patient days of stay were 6.80, 5.54 and 10.47 for 2019, 2020-non-COVID-19 and COVID-19 cases, respectively (*p* < 0.001). HO-CDI episodes per 10,000 patient days of stay were 5.06, 4.63 and 8.13 for 2019, 2020-non-COVID-19 and COVID-19 cases, respectively (*p* < 0.05) (Figure 1 and Table 1).

**TABLE 1:** Admissions, days of hospital stay, and incidence of hospital acquired *Clostridioides difficile* infection in the different study groups.

	2019	2020	
		Non-COVID-19	COVID-19
Admissions	47,048	35,662	6,763
Days of hospital stay (mean)	7.46	8.12	14.75
Total days of hospital stay	350,549	239,232	102,398
HA-CDI episodes	238	159	68
HA-CDI incidence	6.80	5.54	10.47
HO-CDI incidence	5.06	4.63	8.13

COVID-19, coronavirus disease; HA-CDI, hospital acquired Clostridioides difficile infection; HO-CDI, hospital onset Clostridioides difficile infection.

For clinical assessment, we included 138 HA-CDI episodes from 2019, 153 HA-CDI episodes from 2020 without COVID-19 and 68 HA-CDI episodes with COVID-19.

We compared the HA-CDI clinical presentation and evolution between all groups (Tables 2, 3).

**TABLE 2:** Characteristics and clinical data of patients with hospital acquired *Clostridioides difficile* infection with and without coronavirus disease.

	HA-CDI without COVID-19 2019	HA-CDI without COVID-19 2020	HA-CDI with COVID-19	*P*-value
	*N* = 138	*N* = 153	*N* = 68	
**Age** Median (q1, q3)	71.0 (55.0, 82.8)	73.0 (59.0, 85.0)	75.0 (66.0, 84.0)	0.107
>65 years	83 (60.1%)	96 (62.7%)	52 (76.5%)	0.061
**Gender**				0.827
Male	66 (47.8%)	72 (47.1%)	35 (51.5%)	
Female	72 (52.2%)	81 (52.9%)	33 (48.5%)	
Days of hospital stay median (q1, q3)	19.0 (12.5, 35.0)	20.0 (11.0, 39.0)	30.5 (15.8, 57.5)	0.014
**COMORBIDITIES**				
Charlson’s comorbidity index median (q1, q3)	4.0 (3.0, 6.0)	3.0 (2.0, 5.0)	3.0 (2.0, 5.0)	0.034
Malignancy	36 (26.1%)	38 (24.8%)	6 (8.8%)	0.012
Immunosuppressed	60 (43.5%)	44 (28.8%)	28 (41.2%)	0.024

COVID-19, coronavirus disease; HA-CDI, hospital acquired Clostridioides difficile infection.

**TABLE 3:** *Clostridioides difficile* risk factors, episode characteristics, and outcomes in coronavirus disease and no coronavirus disease patients.

	HA-CDI without COVID-19 2019	HA-CDI without COVID-19 2020	HA-CDI with COVID-19	*P*-value
	*N* = 138	*N* = 153	*N* = 68	
**CDI RISK FACTORS**				
Antibiotic treatment	133 (96.4%)	147 (96.1%)	67 (98.5%)	0.628
Third Generation Cephalosporins	54/133 (40.6%)	58/147 (39.5%)	39/67 (58.2%)	0.026
Macrolides	10/133 (7.5%)	13/147 (8.8%)	12/67 (17.9%)	0.057
Proton pump inhibitors use	123 (89.1%)	148 (96.7%)	63 (92.6%)	0.039
Nasogastric tube	27 (19.7%)	29 (19.0%)	13 (19.1%)	0.986
Mechanical ventilation	29 (21.2%)	28 (18.3%)	10 (14.7%)	0.528
Surgery	43 (31.4%)	41 (27.0%)	12 (17.6%)	0.113
**CDI EPISODE**				
Fever	35 (25.4%)	29 (19.0%)	7 (10.3%)	0.036
Days of diarrhea Median (q1, q3)	4.0 (2.0, 6.0)	4.0 (2.0, 7.0)	4.0 (3.0, 5.0)	0.522
Abdominal pain	60/137 (43.8%)	55 (35.9%)	19 (27.9%)	0.077
Toxic megacolon	2/137 (1.5%)	0 (0.0%)	0 (0.0%)	0.197
Pseudomembranous colitis	0 (0.0%)	3 (2.0%)	1 (1.5%)	0.269
**CDI episode severity**				0.029
Mild	94 (68.1%)	90 (58.8%)	46 (67.6%)	
Severe	32 (23.2%)	58 (37.9%)	17 (25.0%)	
Severe-complicated	12 (8.7%)	5 (3.3%)	5 (7.4%)	
Received CDI treatment	132/137 (96.4%)	146 (95.4%)	66 (97.1%)	0.829
Metronidazole treatment	50/132 (37.6%)	28/146 (19.2%)	14/66 (21.2%)	0.001
Vancomycin treatment	110/132 (82.7%)	138/146 (94.5%)	63/66 (95.5%)	0.001
Fidaxomicin treatment	6/132 (4.5%)	3/146 (2.1%)	0/66 (0.0%)	0.147
Fecal microbiota transplantation	6/132 (4.5%)	1/146 (0.7%)	0/66 (0.0%)	0.033
Bezlotoxumab	4/132 (3.0%)	5/146 (3.4%)	1/66 (1.5%)	0.742
Colectomy surgery due to CDI	1 (0.7%)	1 (0.7%)	0 (0.0%)	0.788
**CDI OUTCOMES**				
ICU admissions	6 (4.3%)	2 (1.3%)	2 (3.0%)	0.289
In-hospital death	9 (6.9%)	21 (14.3%)	15 (23.1%)	0.006
CDI-related death	3 (2.17%)	17(11.11%)	8 (11.76%)	0.007
Treatment failure	2/132 (1.5%)	4/134 (3.0%)	4/61 (6.6%)	0.167
Recurrence	23/120 (19.2%)	20/121 (16.5%)	15/53 (28.3%)	0.195

COVID-19, coronavirus disease; HA-CDI, hospital acquired Clostridioides difficile infection; ICU, intensive care unit.

We found no significant differences between groups for sex and age. The median Charlson comorbidity index at admission was 3.0 (Q1-Q3: 2.0–5.0) in both COVID-19 and non-COVID-19 groups of 2020 and 4.0 (Q1-Q3: 3.0–6.0) in the 2019 group (*p* = 0.034) (Table 2).

HA-CDI cases with COVID-19 had longer hospital stays and a lower incidence of malignancies (*p* < 0.05) than those in the other groups. The proportion of immunosuppressed patients prior to admission was lower in the 2020 non-COVID-19 group (*p* = 0.024).

Concerning to the use of antibiotics in the previous month to the CDI episode, overall, there were no significant differences between all groups (96.4% HA-CDI 2019, 96.1% HA-CDI non-COVID-19 and 98.5% HA-CDI-COVID-19). In both years the number of defined daily doses (DDDs) were not significantly different, from 148,740 DDD per 100 hospital stays (DDD per 100 HS) in 2019 to 143,157 DDD per 100 HS in 2020 (*p*-value 0.39). However, third generation cephalosporins use was significantly higher in the COVID-19 group than in non-COVID-19 groups (*p* = 0.026) (Table 3).

Another CDI risk factors such as proton pump inhibitor use in the previous month was higher in the HA-CDI-2020 non-COVID group (96.7%), followed by HA-CDI-COVID group (92.6%) and HA-CDI 2019 group (89.1%) (*p* = 0.039). No significant differences were found in the presence of other CDI risk factors in the month prior to the CDI episode, such as the use of nasogastric tube, mechanical ventilation or surgery.

No significant differences were seen for days of diarrhea, toxic megacolon, and demonstration of pseudomembranous colitis between study groups. Overall, most of the CDI episodes were mild. CDI episodes were severe in, 23.2%, % of HA-CDI 2019 patients, 37.9%% in HA-CDI-2020 without COVID-19 and 25.0% in HA-CDI COVID-19 patients (*p* = 0.014).

Regarding CDI treatment, vancomycin was the most prescribed antimicrobial (82.7% HA-CDI 2019, 94.5% HA-CDI non-COVID-19 and 95.5% HA-CDI-COVID-19), followed by metronidazole (37.6% HA-CDI 2019, 19.2% HA-CDI non-COVID-19 and 21.2% HA-CDI-COVID-19). There was an increase in the use of vancomycin in detriment of metronidazole use during 2020 with respect to 2019 (*p* = 0.001) (Table 3). There were no significant differences in the use of fidaxomicin and bezlotoxumab. As for fecal transplantation, only one procedure was performed during the 2020 pandemic in HA-CDI patients.

Surgical treatment of CDI colonic disease was distinctly uncommon with no difference between the study groups.

Overall, in-hospital death was significantly higher in HA-CDI COVID-19 patients (23.1%) than in HA-CDI 2019 patients (6.9%) and in HA-CDI-2020 without COVID-19 (14.3%) (*p* = 0.006). When we tried to approach potential HA-CDI causality in the cause of death, we found mortality potentially related to CDI to be higher in 2020 with respect to HA-CDI cases in 2019 (*p* = 0.007), however there was no significant differences between HA-CDI-2020 without COVID-19 and HA-CDI COVID-19 patients (*p* = 0.88) (Table 3).

CDI treatment failure was 1.5, 3, and 6.6% of HA-CDI 2019 group, HA-CDI-2020 without COVID-19 and HA-CDI COVID-19, respectively (*p* = 0.167). Finally, CDI recurrence occurred in 19.2, 16.5, and 28.3% of HA-CDI 2019 group, HA-CDI-2020 without COVID-19 and HA-CDI COVID-19, respectively (*p* = 0.195) (Table 3).

## Discussion

Our results show that in the population of patients with COVID-19, incidence of both HA-CDI and HO-CDI was higher than in non-COVID-19 cases. Regarding clinical characteristics of the CDI episode there were hardly any significant differences in the COVID-19 patients with respect to non-COVID patients, although there was a trend toward higher risk of CDI recurrence in COVID-19 patients.

The COVID-19 pandemic in our hospital started on March 1, 2020. It led to massive care of patients who needed special isolation measures and received more broad-spectrum antibiotics for fear of possible bacterial co-infection as reported by many other institutions (13, 14).

We found a significant increase in the use of third-generation cephalosporins in COVID-19 cases, which are a well-known risk factor for CDI (15). In addition, patients with COVID-19 have been reported to suffer from dysbiosis of the gut microbiota, similar to that caused by antibiotic use, a major risk for CDI infection (16).

Data on the incidence of HA-CDI in COVID-19 patients is still scarce and disparate (5–8, 14, 17–21) several studies describe a lower incidence of CDI during the pandemic (5–8). As summarized by Granata et al., most studies find a lower or equal incidence of CDI in the first phase of the pandemic than in the pre-pandemic period (19). However, in the majority of these studies, the used denominators are the days of hospital stay of the whole population and not only the COVID-19 cases.

There was a CDI incidence in COVID-19 patients in our hospital that falls within the ranges that have been observed in other hospitals (18).

Lewandowski et al. reported an increase in HA-CDI episodes, as in our study. The authors included the population of COVID-19 patients and as denominators the days of hospital stay of the COVID-19 cases (4).

Our comparison of the HA-CDI in non-COVID-19 cases between the 2019 and 2020 episodes indicate that the difference in incidence cannot be attributed to the changes of hospitals’ hygiene control measures. E. Bentivegna et al. reported a decrease of CDI in COVID-19 free units, while in COVID-19 units the incidence of CDI increased (22).

In our opinion, a paradoxical effect in the incidence of HA-CDI does not occur and the increase in the HA-CDI incidence in COVID-19 patients is concordant with the presence in this population of risk factors such as long stay and broad-spectrum antibiotic use.

Our data suggest higher recurrence rate of HA-CDI episodes and CDI treatment failure in COVID-19 patients, which should motivate a less restrictive use of drugs that showed already an ability to reduce recurrences as registered in recent guidelines (10, 23). Regarding CDI disease severity, COVID-19 does not seem to have a profound impact. During the 2020 pandemic, HA-CDI patients without COVID had more frequently severe disease than HA-CDI-2020 with COVID-19, possibly due to the fact that during the pandemic in 2020 only patients with a worse state of health sought care at the hospital.

The attribution of mortality in HA-CDI episodes is complicated; we were unable to demonstrate significant higher HA-CDI-related mortality in COVID-19 cases. Nevertheless, we did find a higher HA-CDI-related mortality in patients in the pandemic period, both with COVID-19 and without COVID-19 compared to HA-CDI patients in 2019.

The main limitation of this study is the single-centre design, but with long-term interest in the topic.

Limiting antimicrobial use in COVID-19 cases, shorter hospital stays, and better prevention are the main stay of HA-CDI prevention.

## Data availability statement

The raw data supporting the conclusions of this article will be made available by the authors, without undue reservation.

## Ethics statement

The studies involving human participants were reviewed and approved by the Ethics Committee of Hospital General Universitario Gregorio Marañón in Madrid. Written informed consent from the participants’ legal guardian/next of kin was not required to participate in this study in accordance with the national legislation and the institutional requirements.

## Author contributions

SV-C, ER, and EB designed the study and had access and verified the underlying study data. SV-C performed the collection of data and data analysis and wrote the first draft of the manuscript. All authors contributed to data interpretation, contributed with the intellectual content and approved the final draft, and had full access to all the data in the study and had final responsibility for the decision to submit for publication.
